# Supplementary material for: Overpumping leads to California groundwater arsenic threat
Source: Nat Commun. 2018 Jun 5;9:2089. doi: 10.1038/s41467-018-04475-3 (PMC5988660; doi:10.1038/s41467-018-04475-3)
Supplement: Supplementary file 1 — Supplementary Information [file 41467_2018_4475_MOESM1_ESM.pdf]

Nature Communications

Supplementary Information for

Over-Pumping Leads to California Groundwater Arsenic Threat

Smith et al., 2018

Supplementary Table 1: List of variables, their short names, and their sources

Variable name	Variable description	Source
corc.thick	Thickness of Corcoran clay, m	[1]
elev	Land surface elevation, m	[2]
log10_mn	log10(Mn concentration, ppb)	[3]
insar	Mean subsidence velocity, cm/yr, 2007 to 2011	See methods
loncv	Longitude, degrees	
pct.gw	Percent of total water usage that is groundwater	[4]
clay	Total thickness of fine-grained deposits, m	[4]
log10_so4	log10(SO4 concentration, ppb)	[3]
perfb	Bottom of perforated interval, m	[4]
latcv	Latitude, degrees	
et.pre07	Estimated evapotranspiration, pre-2007, m/yr	[5]
Q	Estimated historic groundwater flow, m/day	See methods
avgT	Average temperature over the past 30 years, degrees C	[6]
sub76	Historical subsidence from 1960 to 1976, cm/yr	[4]
sub86to93	Historical subsidence from 1986 to 1993, cm/yr	[4]
et.post07	Estimated evapotranspiration, post-2007, m/yr	[5]
driv	Distance from nearest river, m	See methods
perft	Top of perforated interval, m	[4]

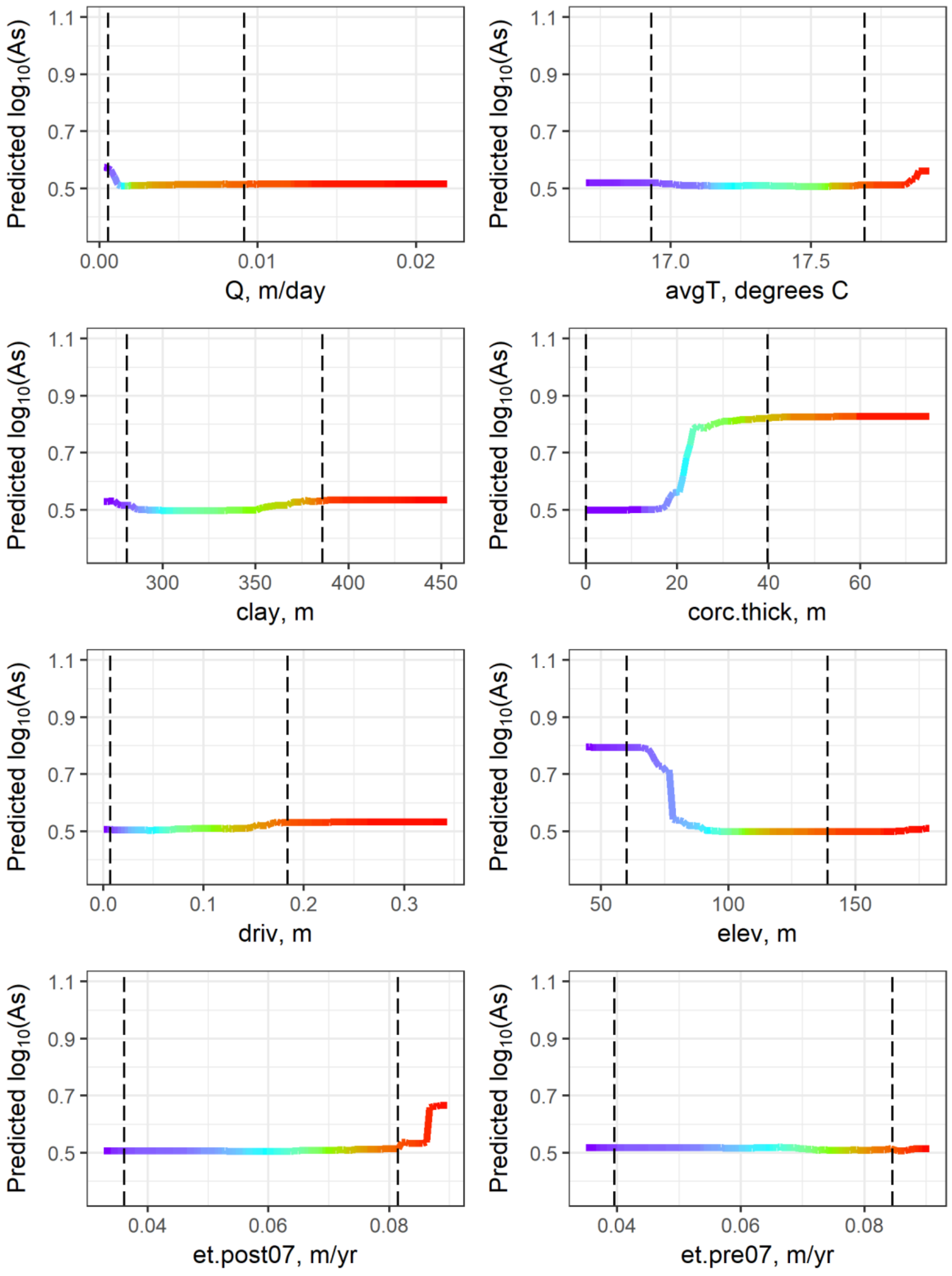
Predicted log10(As)
1.1
0.9
0.7
0.5
0.00
0.01
0.02
Q, m/day
17.0
17.5
avgT, degrees C
300
350
400
450
clay, m
0
20
40
60
corc.thick, m
0.0
0.1
0.2
0.3
driv, m
50
100
150
elev, m
0.04
0.06
0.08
et.post07, m/yr
et.pre07, m/yr

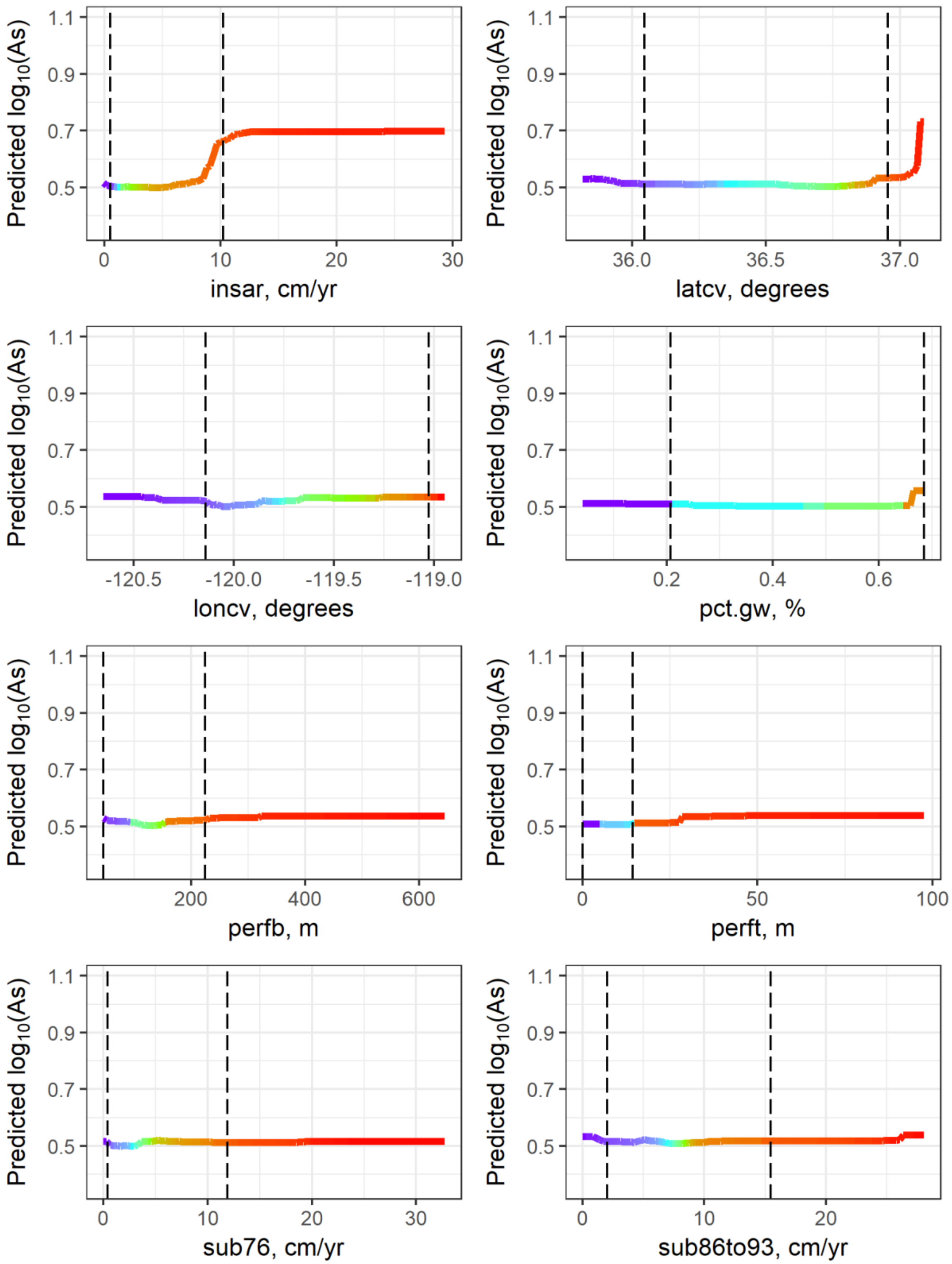

Predicted log_{10}(As)
1.1
0.9
0.7
0.5
0
10
20
30
insar, cm/yr
36.0
36.5
37.0
latcv, degrees
-120.5
-120.0
-119.5
-119.0
loncv, degrees
0.2
0.4
0.6
pct.gw, %
200
400
600
perfb, m
0
50
100
perft, m
0
10
20
30
sub76, cm/yr
0
10
20
sub86to93, cm/yr

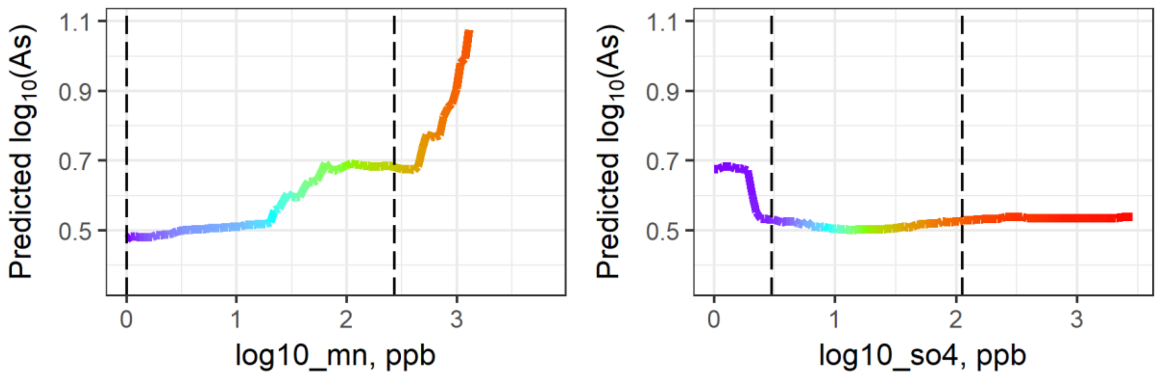

Supplementary Figure 1: Partial dependence plots. These are calculated for all variables used in the model predicting arsenic concentrations from 2007-2015. Color shows the percentile of the data. Dashed lines at the 5th and 95th percentiles.

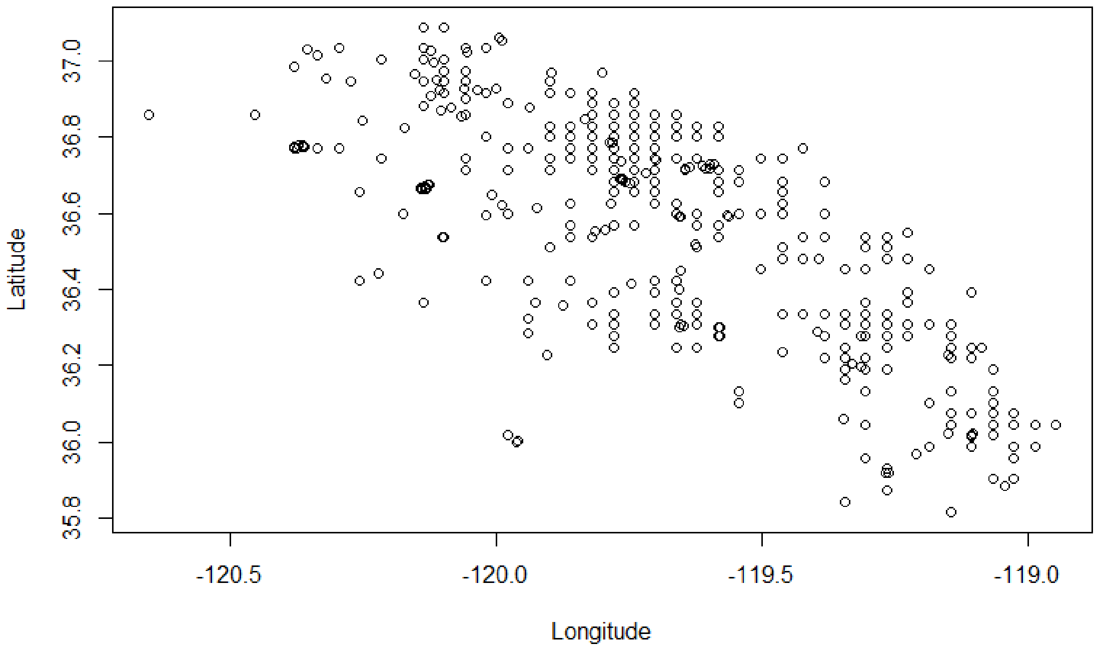

Supplementary Figure 2: Locations of public supply wells. These wells contain arsenic, manganese and sulfate concentration data between 2007 and 2015.

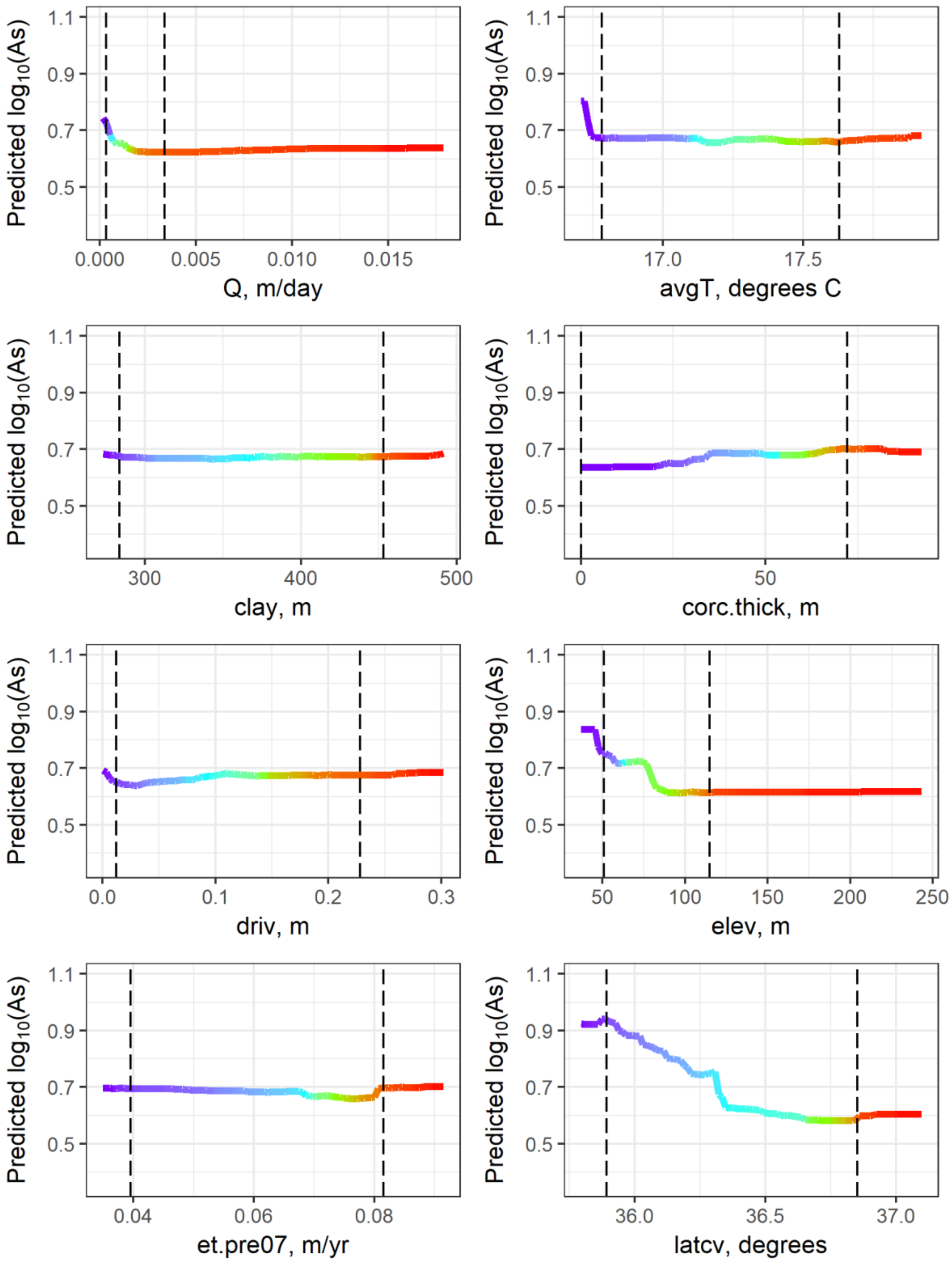
Predicted log10(As)
Q, m/day
avgT, degrees C
clay, m
corc.thick, m
driv, m
elev, m
et.pre07, m/yr
latcv, degrees

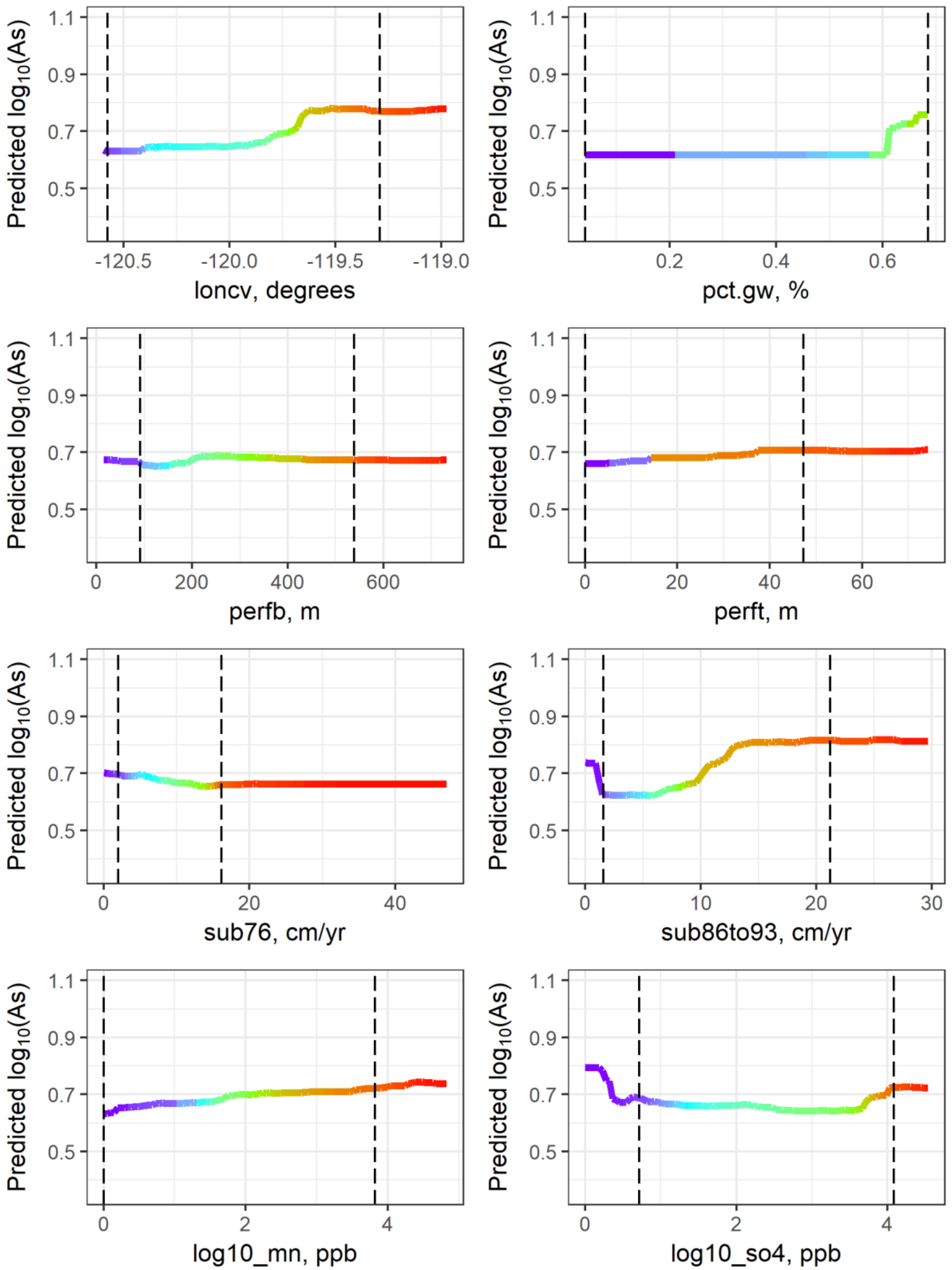

Supplementary Figure 3: Partial dependence plots. These are calculated for all variables used in the model predicting arsenic concentrations from 1986-1993. Color shows the percentile of the data. Dashed lines at the 5th and 95th percentiles.

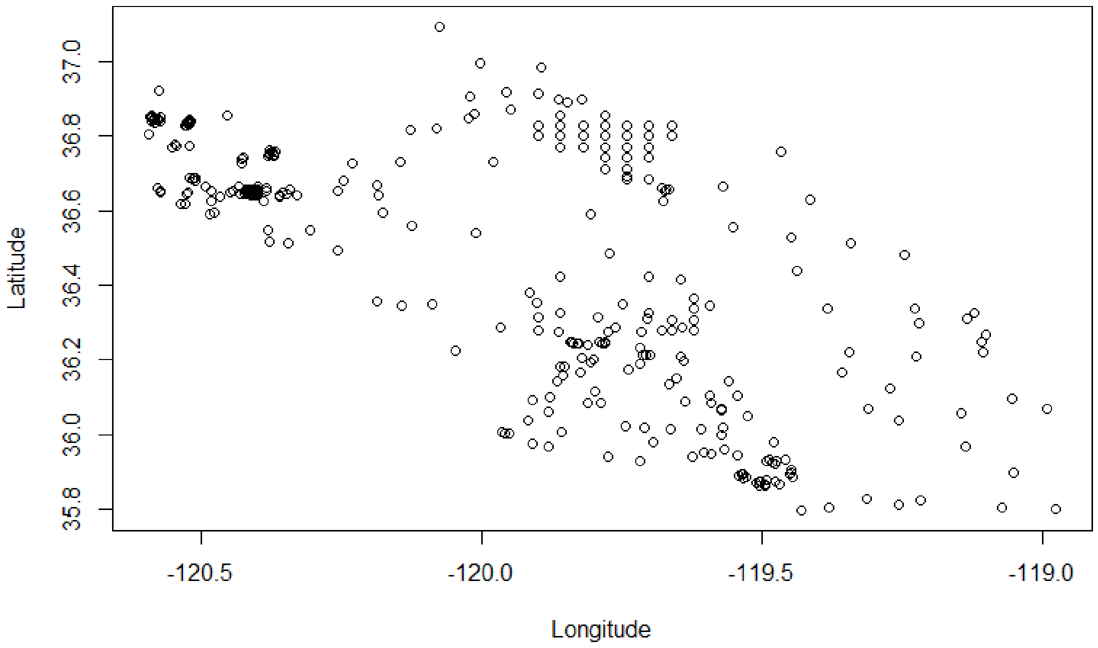

Supplementary Figure 4: Locations of public supply wells. These wells contain arsenic, manganese and sulfate concentration data between 1986 and 1993.

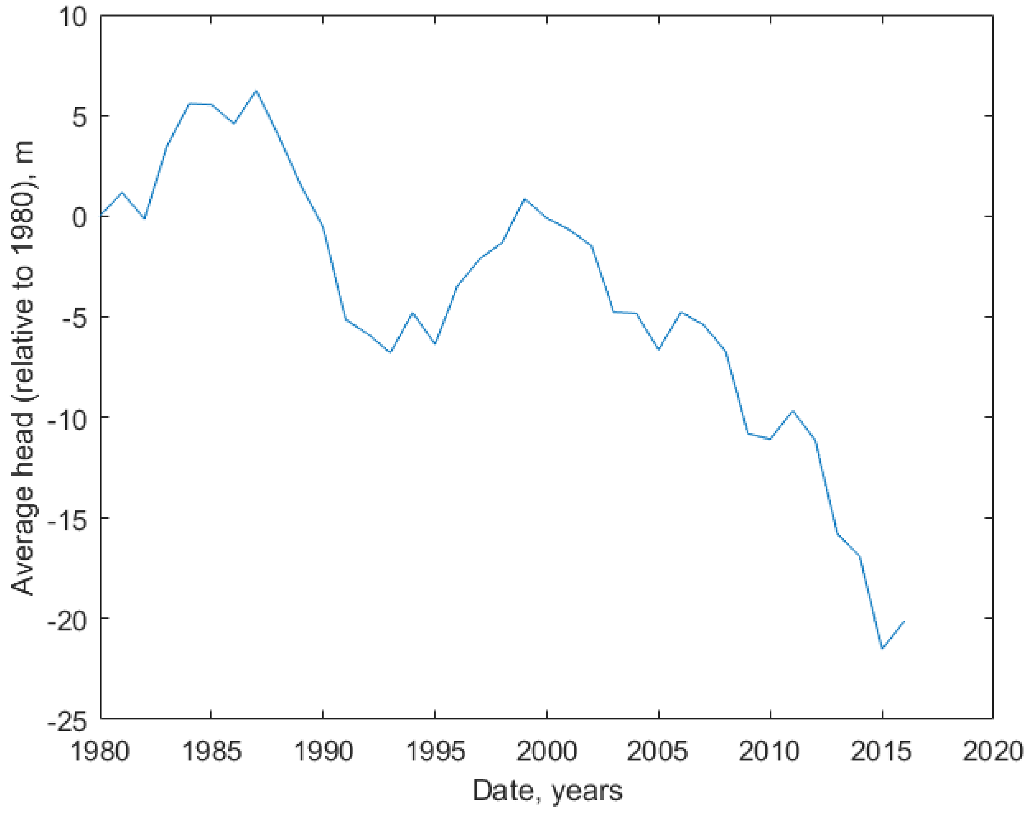

Supplementary Figure 5: Average head. Measured in the spring, relative to 1980 spring heads for all wells in study area containing spring head data in 1980. Note that the 1986-1993 drought produced a ~11 m drop in head, while the 2007-2015 combined droughts produced a ~16 m drop in head.

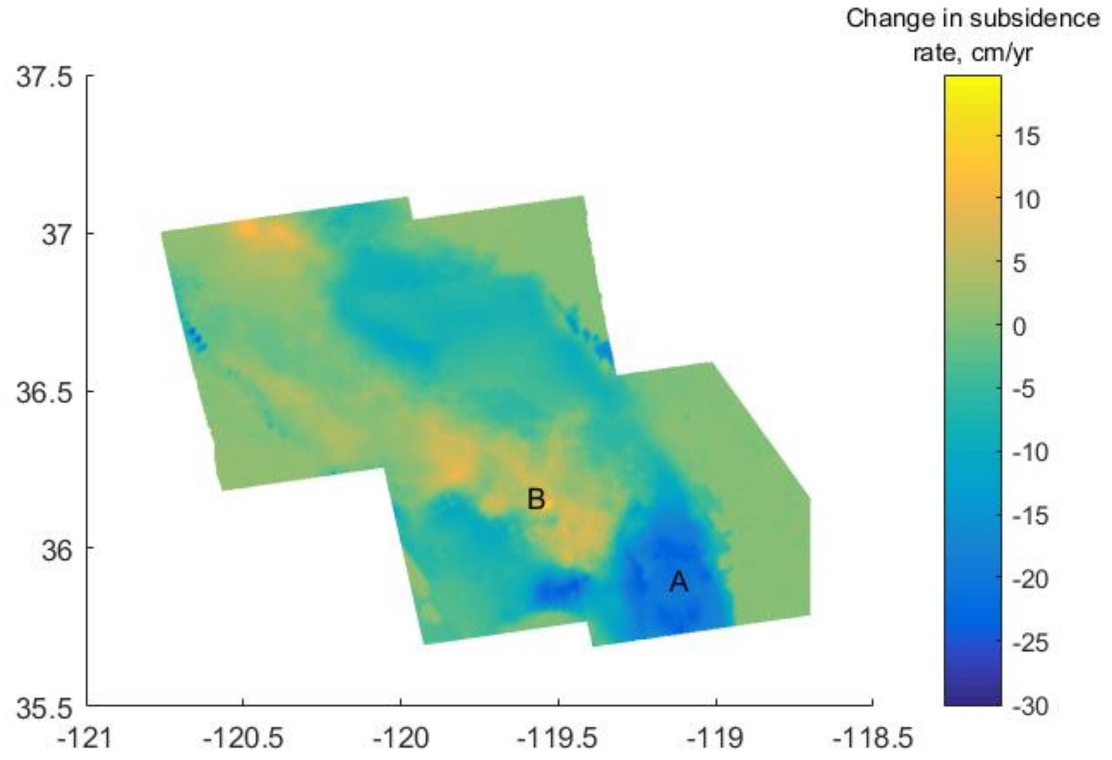

Supplementary Figure 6: Change in subsidence rate. Calculated by subtracting the subsidence rate from the 1986-1993 period (modeled by Faunt et al., 2009) from the 2007-2010 period (mapped by InSAR). Positive values indicate an increase in subsidence over time. Note the shift in subsidence from the southeast part of the valley from 1986-1993 (**A**) to a more central part of the valley from 2007-2010 (**B**). Subsidence from 2012-2015 follows a similar spatial pattern to that from 2007-2010 (Farr and Liu, 2015).

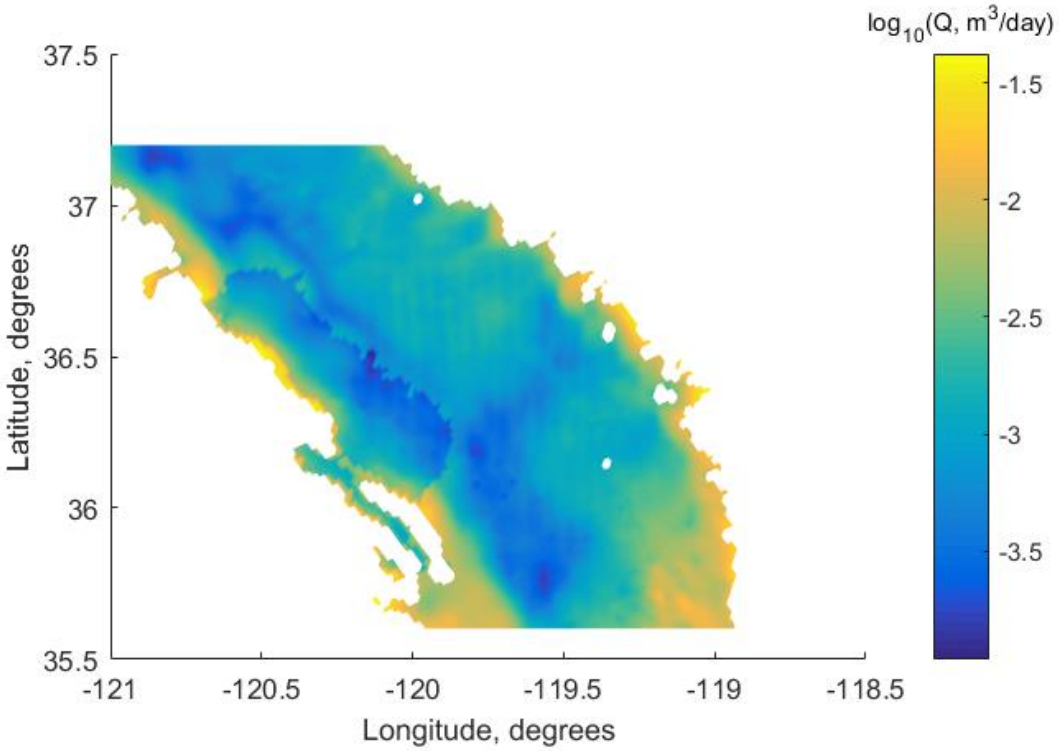

Supplementary Figure 7: Estimated historic groundwater flow. Units are in m/day (flow per m^2 cross-sectional area). The color scale is logarithmic. This dataset was created by multiplying the land surface slope by the estimated hydraulic conductivity from Faunt et al. (2009), where both datasets exist.

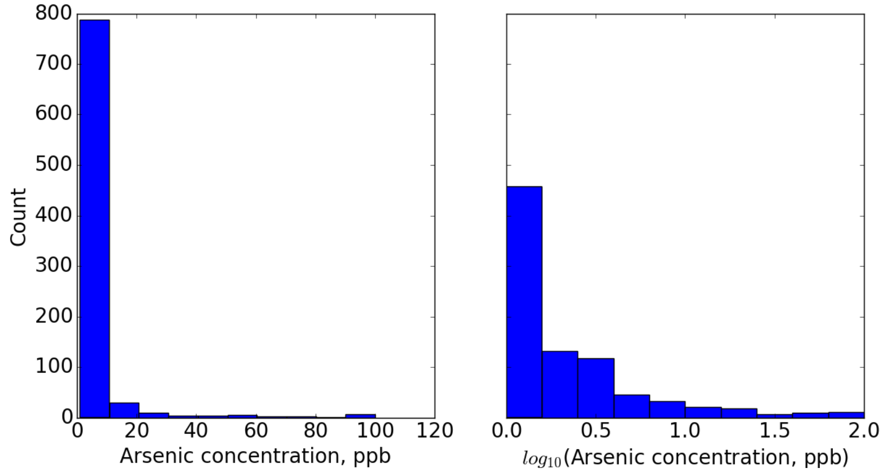

Supplementary Figure 8: Arsenic concentration histograms. This illustrates the value of using logarithmic values for arsenic concentrations (right) over the traditional

approach (left). Note that taking the logarithm accounts for the significant differences in magnitude in concentration measurements.

References

[1] Page, R.W. Geology of the Fresh Ground-Water Basin of the Central Valley, California, with Texture Maps and Sections. *U.S. Geol. Surv. Prof. Pap.* **1401-C**, 1-54 (1986).

[2] Farr, T.G., Rosen, P.A., Caro, E., Crippen, R., Duren, R., Hensley, S., Kobrick, M., Paller, M., Rodriguez, E., Roth, L. and Seal, D. The shuttle radar topography mission. Reviews of geophysics, **45(2)**, 1-33 (2007).

[3] Belitz, K., Dubrovsky, N. M., Burow, K., Jurgens, B., & Johnson, T. Framework for a ground-water quality monitoring and assessment program for California. *US Department of the Interior, US Geological Survey* (2003).

[4] Faunt, C. C., R. T. Hanson, K. Belitz, W. Schmid, S. P. Predmore, D. L. Rewis, and McPherson, K. Groundwater Availability of the Central Valley Aquifer, California. *U.S. Geol. Surv. Prof. Pap.*, **1776**, 1-225 (2009).

[5] Anderson, M.C., Norman, J.M., Mecikalski, J.R., Otkin, J.A. and Kustas, W.P. A climatological study of evapotranspiration and moisture stress across the continental United States based on thermal remote sensing: 1. Model formulation. *Journal of Geophysical Research:Atmospheres*, https://agupubs.onlinelibrary.wiley.com/doi/epdf/10.1029/2006JD007506 (2007).

[6] Daly, C., Neilson, R., and Phillips, D. A statistical-topographic model for mapping climatological precipitation over mountainous terrain. *Journal of applied meteorology* **33(2),** 140-158 (1994).
